# Direct versus referred admission to the maternity hospital due to preeclampsia: Does it influence pregnancy outcomes?

**DOI:** 10.1002/ijgo.70772

**Published:** 2026-01-05

**Authors:** Juliana da‐Costa‐Santos, Guilherme F. Borges, Vitor L. V. Reis, Rodolfo R. Japecanga, Maykon A. Carvalho, Ana Julia G. Oliveira, Laura C. Vinchi, Arthur B. P. Righi, Jussara Mayrink, Fernanda G. Surita, José Paulo de Siqueira Guida, Renato T. Souza, José G. Cecatti, Maria Laura Costa

**Affiliations:** ^1^ Department of Obstetrics and Gynecology, School of Medical Sciences Universidade Estadual de Campinas Campinas Brazil; ^2^ Pontifical Catholic University of Campinas Campinas Brazil; ^3^ Department of Obstetrics and Gynecology, School of Medicine Federal University of Minas Gerais Belo Horizonte Brazil

**Keywords:** delivery of health care, perinatal care, preeclampsia, pregnancy complications, referrals

## Abstract

**Objectives:**

This study describes maternal and perinatal outcomes of women with preeclampsia (PE) according to hospital admission characteristics: referral from lower complexity settings or directly admitted at the reference hospital.

**Methods:**

This is a cross‐sectional study considering women with PE who were admitted for childbirth between September 2019 and August 2021, at a Brazilian university hospital. Women were divided into hospitalized at the tertiary center or referred women (from facilities in the region). The main outcome measures were need for magnesium sulfate (proxy for severity), mean gestational age (GA) at childbirth, 5‐min Apgar score <7, birthweight, and maternal and perinatal deaths.

**Results:**

There were 3914 childbirths, with 428 with PE (10.9%). Among PE cases, 37 (8.6%) were referred cases. Magnesium sulfate was used in 89.2% of the referred women and 44.8% of non‐referred (*P* < 0.001). The mean GA at childbirth differed significantly between groups (referred cases: 33.6 ± 3.27 weeks vs. 36.1 ± 2.97 weeks, *P* < 0.001). Five‐minute Apgar <7 was more frequent among referred cases (13.9% vs. 4.1%, *P* = 0.03). There were no maternal deaths and no differences in perinatal deaths between groups. Newborns from referred mothers had lower mean birthweight (2150 ± 946 vs. 2730 ± 800 g, *P* < 0.001) and needed more intensive care (72.2 vs. 34.9%, *P* < 0.001).

**Conclusion:**

Referred women with PE had worse maternal and perinatal outcomes than those who were directly hospitalized in the tertiary center, which highlights the importance of timely diagnosis and delays associated to referral of women with PE.

## 1 INTRODUCTION

Preeclampsia (PE) is a prevalent condition, occurring in 2%–4% of the pregnancies globally. It disproportionately affects pregnant women from low‐ and middle‐income countries (LMIC) such as Brazil, where the estimated prevalence of PE is 6.7% and PE is the leading cause of maternal mortality.^1^, ^2^, ^3^ PE is defined as hypertension after 20 weeks of gestational age (GA) and proteinuria or target‐organ damage (thrombocytopenia, elevation of liver transaminases, renal injury, pulmonary edema, symptoms of imminent eclampsia, or eclampsia) or placental dysfunction characterized by fetal growth restriction and/or compromised fetal Doppler.^2^, ^4^ PE with severe features includes severe hypertension and/or target‐organ damage, which suggest imminent eclampsia, HELLP syndrome (hemolysis, elevated liver enzymes, and low platelets), or eclampsia itself.^4^

After the diagnosis of PE, inpatient management is recommended by the International Society for the Study of Hypertension in Pregnancy (ISSHP) and the national protocols.^2^, ^4^ When there are severe features, urgent care is needed with magnesium sulfate administration for prevention of seizures, along with maternal and fetal surveillance, which will require resources that are not always easily available everywhere. These resources involve trained staff and adequate supplies. Therefore, referral is a life‐saving asset that allows for timely and adequate management of PE. As per the World Health Organization, a high value referral aims to provide the best care for the patient within the limitations of the health system, while eliminating unnecessary interventions.^5^ In Brazil, the public health system's coordination between different healthcare facilities integrates and distributes the health demands according to the levels of complexity.^6^ This access regulation system is also responsible for directing pregnant women to an adequate facility to manage pregnancy complications such as PE. The referrals rely on the correct clinical suspicion or diagnosis at the lower complexity facilities, timely evaluation of each case by the healthcare regulators, availability of resources at the reference center that admits the case, availability of transportation, and management at the higher complexity setting.

Ideally, this should be a quick process. However, in clinical practice, there are three main causes of delayed care, especially in LMICs: difficulty in deciding to seek care, hindered access to healthcare facilities, and delayed access to the correct healthcare.^7^ In Brazil, a study involving over 82 000 women found that any kind of delay was observed in 53.8% of all subjects. Importantly, the greater the severity, the more delays, with delays for 52.0% of women with potentially life‐threatening conditions, 68.4% with maternal near misses, and 84.1% for maternal deaths.^8^ Lower schooling levels that make it more difficult to understand which signs and symptoms should prompt further evaluation, an imbalance between demand for health care and obtainable resources, and low availability of life‐saving items such as magnesium sulfate are examples of situations that could result in delays.^9^, ^10^

Because PE is a prevalent condition but advanced healthcare resources are limited, the objectives of the current study are to investigate if the maternal and perinatal outcomes vary with distinct modalities of hospitalization and if there are any identified delays. These data can potentially guide interventions to improve maternal and perinatal health.

## 2 MATERIALS AND METHODS

A secondary analysis of an observational cross‐sectional study with a convenience sample was conducted at the University of Campinas (UNICAMP) Women's Hospital, Brazil, between September 1, 2019, and August 31, 2021. This tertiary hospital is located in a metropolitan region, with over 3 million inhabitants, and serves this area and adjacent municipalities outside the metropolitan region. It is a hospital fully dedicated to women's health, equipped with nurseries and intensive care units for obstetrics and neonatology, in which women who are cared for at high‐risk antenatal care outpatient clinics or that spontaneously seek health care in emergency rooms may be hospitalized when necessary. The primary study is published elsewhere.^11^

In the public health system, referral of urgent cases is possible within the healthcare access coordination system via electronic request forms. When primary care units or less complex hospitals request a referral, the regulation center locates and contacts the nearest hospital with sufficient resources to manage the case based on a prespecified geographical distribution.^12^, ^13^ Then, the reference hospital can accept the referral or not, depending on the availability of hospital beds.

In this study, medical records of any pregnant women admitted to childbirth at the institution were reviewed to identify those with PE and, among them, which ones were referred from other healthcare facilities and which ones' hospitalizations were indicated by the assisting team of the university hospital (either those that spontaneously seek care at the emergency rooms or those coming from the low‐ and high‐risk local outpatient clinics). The group “direct admissions” is defined as women who were hospitalized after an outpatient appointment at the institution, either straight from the clinic or after emergent care. The group “external referrals” is defined as women who were initially admitted at lower complexity facilities and then transferred to the reference center. The diagnosis of PE was based on the ISSHP guidelines.^4^ PE with severe features was characterized pragmatically for the purpose of the study using magnesium sulfate to prevent seizures.

Data collected include maternal social and demographic characteristics, obstetric history, pregnancy evolution, moment of diagnosis of PE, moment of diagnosis of PE with signs of severity and GA when applicable, magnesium sulfate use, GA at childbirth, route of delivery, birthweight, 5‐min Apgar scores, and admission to the neonatal intensive care unit (ICU), among others. A specific section of the data collection of the referred cases was dedicated to the information regarding maternal and fetal conditions at the first approach in the lower complexity setting, which municipality they came from, and time from request of referral to arrival at the tertiary center. Finally, because the period studied involves the COVID‐19 pandemic, this was considered a co‐variable. Confirmed COVID‐19 during pregnancy was considered when a positive molecular test (qRT‐PCR or antigen test) was obtained.

Qualitative variables were described as frequencies and percentages, while quantitative variables were described as means and standard deviations. The association of variables was tested with the *χ* ^2^‐test or Fisher's exact test for categorical variables, or the *t*‐test or Mann–Whitney test according to the distribution of quantitative variables. Finally, a composite variable called “any adverse maternal outcome” was elaborated using route of birth, use of magnesium sulfate, eclampsia, and maternal death. This variable was involved in a binary multivariate logistic regression, performed to determine which variables were independent predictors of adverse maternal outcomes.

Data were stored in a Microsoft Excel file, access to which was restricted to the data collectors and had specific codification to ensure anonymity. Statistical analysis was conducted in the software R version 4.3.0 and involved descriptive and inferential analyses to compare women who were referred and standard admissions.

In case of missing data, the amount of unknown information is declared below each table, and the comparison was made excluding the missing information. Quantitative variables were described in mean and standard deviations, while qualitative variables were expressed as absolute numbers and percentages. The association of categorical variables was evaluated with the *χ* ^2^‐test or Fisher's exact test, while numerical variables were analyzed with the *t*‐test or the Mann–Whitney test. For bivariate analyses, *P*‐values <0.05 were considered statistically significant. Figures were generated with the software R version 4.3.0.

This study follows the principles of the Declaration of Helsinki. Ethical approval was obtained from the institutional ethical review board prior to any data collection (approval #60249222.3.0000.504). Data confidentiality was assured, and informed consent was waived, but the ethical review board required a term for utilization of these data, formalizing safety and anonymity of patient information. The current report follows the Strengthening the Reporting of OBservational studies in Epidemiology (STROBE) guidelines.^14^

## 3 RESULTS

**FIGURE 1:**
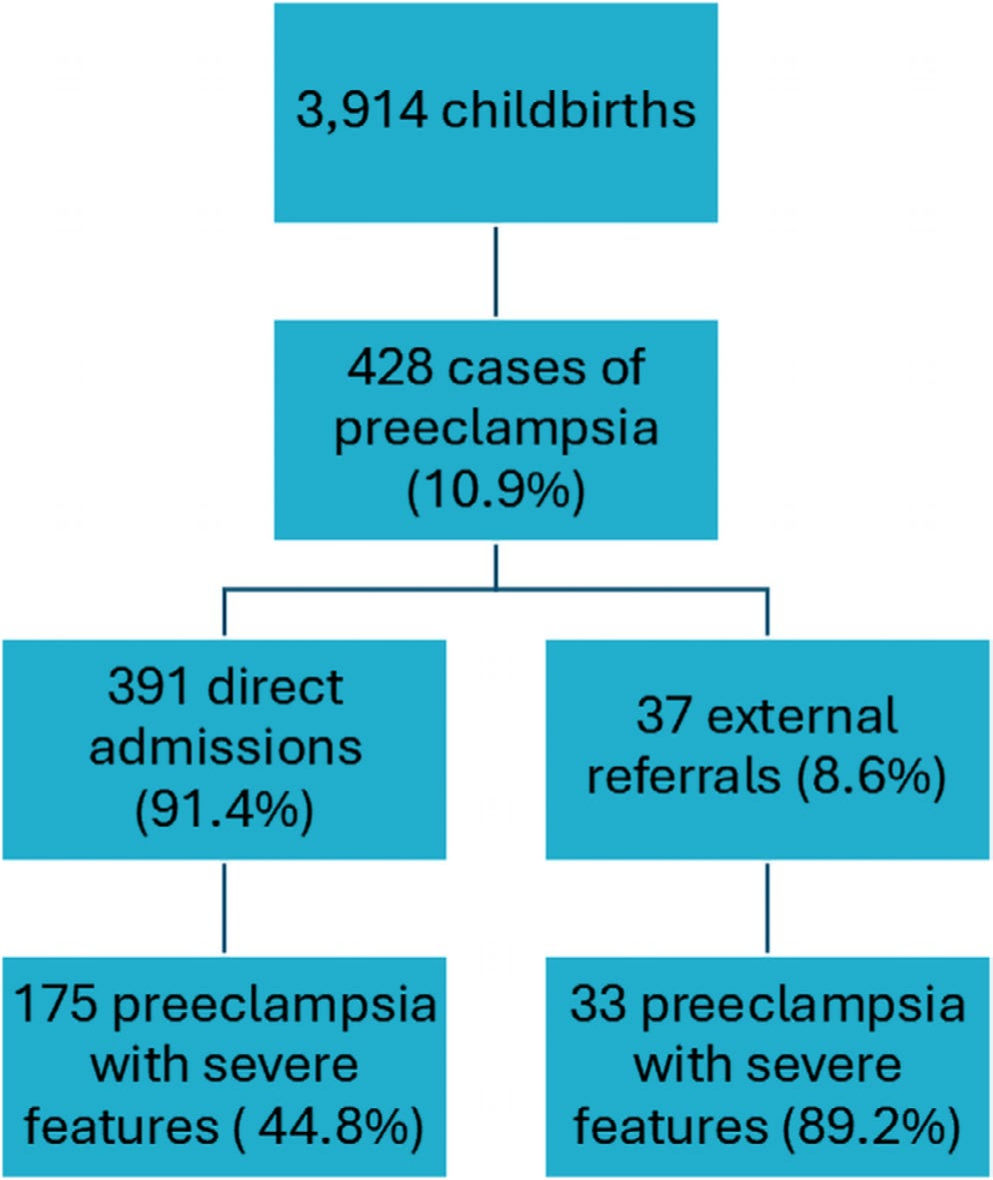
Flow chart of participant selection. There were 3914 childbirths from September 1, 2019, to August 31, 2021. Preeclampsia was diagnosed if hypertension and proteinuria and/or end‐organ damage were present. Severity was considered if magnesium sulfate was indicated to prevent seizures.

There were overall 3914 childbirths, among which 428 with a diagnosis of PE (prevalence of 10.9%). Among women with PE, 37 (8.6%) were external referrals (Figure 1).

**FIGURE 2:**
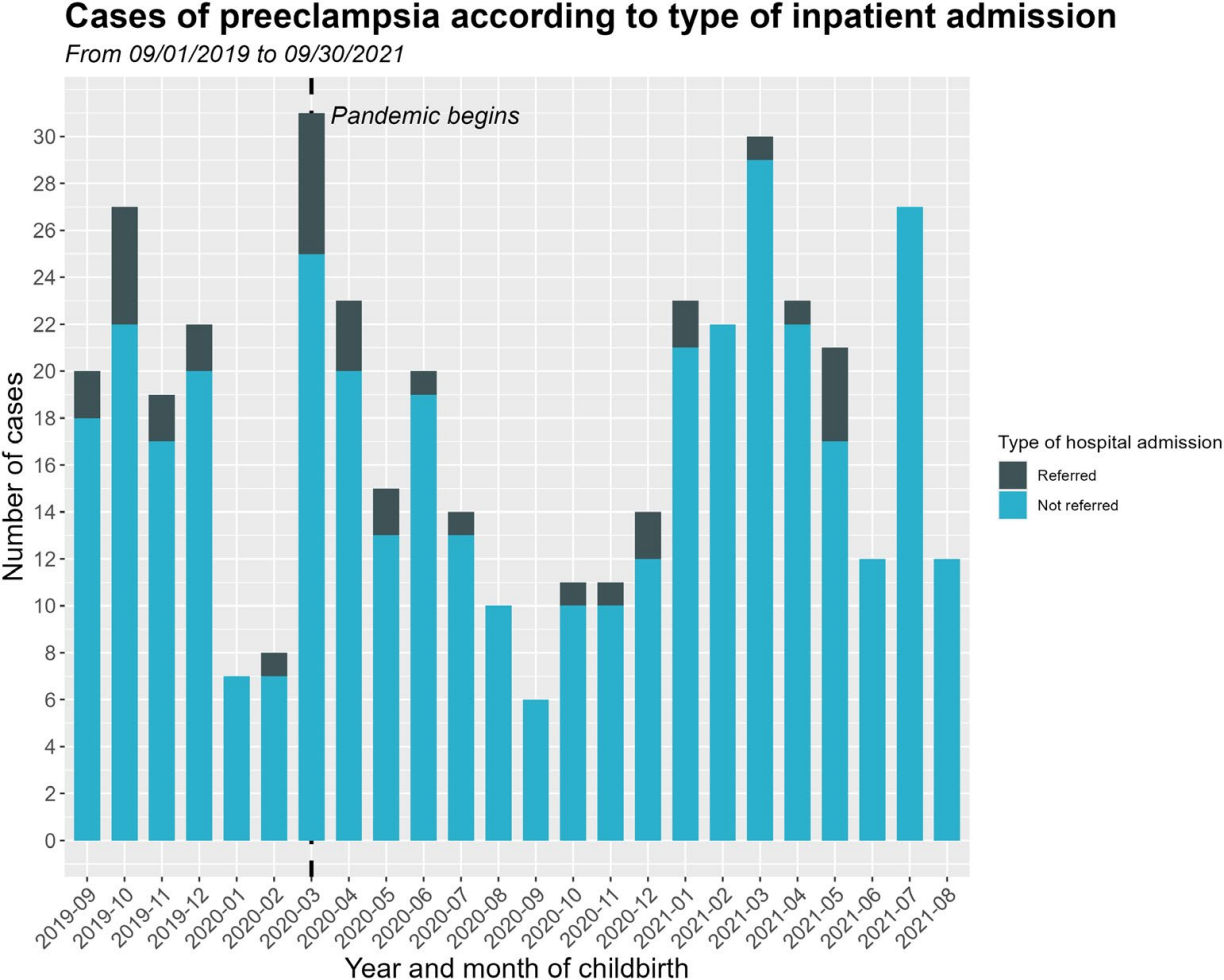
Bar chart showing the absolute frequencies of hospital admissions after referral or not, according to year and month of occurrence, from September 2019 to August 2021.

Social and demographic characteristics, obstetric history and clinical background were similar between women with preeclampsia who were directly admitted or referred from other centers. The women were around 30 years old, mostly with white referred skin color, multiparous, and with a partner, and nearly 30% of them had underlying chronic hypertension (Tables 1 and 2). Women in the direct admissions group had diabetes significantly more often (18.9% vs. 41.3%, *P =* 0.01). Few cases presented confirmed COVID‐19 in the period considered (Table 2). Further, the pandemic had no statistical association with the frequency of referrals; however, it did affect the overall number of deliveries in the period, reducing low‐risk admissions (Figure 2).

**TABLE 1:** Social and demographic characteristics of pregnant women included in the study.

Sociodemographic characteristics	External referrals	Direct admissions	*P*‐value
	(*N =* 37)	(*N =* 391)	
Mean maternal age (SD)	32.2 (8.02)	30.2 (6.79)	0.07
Skin color^a^			
White	20 (60.6%)	246 (63.6%)	0.78
Pardo	11 (33.3%)	108 (27.9%)	
Black	2 (6.1%)	33 (8.5%)	
Marital status			
With a partner	30 (81.1%)	272 (69.6%)	0.20
Educational level^b^			
Up to high school	31 (86.1%)	323 (83.9%)	0.91
Higher education or postgraduation	5 (13.9%)	62 (16.1%)	
Paid work during pregnancy^c^	25 (73.5%)	241 (64.3%)	0.37
Smoking	3 (8.1%)	28 (7.2%)	0.74
Alcohol consumption	1 (2.7%)	20 (5.1%)	1.00

*Note*: Missing: ^a^8, ^b^7, ^c^19.

Abbreviation: SD, standard deviation.

**TABLE 2:** Obstetric history and pregnancy characteristics of PE cases, considering admission and referral to hospital.

Obstetric characteristics	External referrals	Direct admissions	*P*‐value
	(*N =* 37)	(*N =* 391)	
Parity			
1	11 (29.7%)	138 (35.3%)	0.68
2	9 (24.3%)	101 (25.8%)	
≥3	17 (45.9%)	152 (38.9%)	
Use of antihypertensive drugs during pregnancy	24 (64.9%)	217 (55.5%)	0.36
Chronic hypertension	9 (24.3%)	129 (33.0%)	0.37
Diabetes^a^	7 (18.9%)	161 (41.3%)	0.01
Confirmed COVID‐19 during pregnancy^b^	2 (5.4%)	18 (4.6%)	0.69
Singleton pregnancy	36 (97.3%)	365 (93.4%)	0.50

*Note*: Missing: ^a^1.

^b^ Confirmed COVID‐19 during pregnancy was considered when a positive molecular test (qRT‐PCR or antigen test) was obtained.

Table 3 shows that over 90% of the diagnoses of PE in both groups were performed during pregnancy or childbirth. PE with severe features was mostly identified during pregnancy at around 34 weeks of GA in both groups. The frequency of PE with severe features was twice as high among external referrals than the direct admissions group (89.2% vs. 44.8%, *P* < 0.001). Eclampsia was 10 times more prevalent among external referrals (5.4 vs. 0.5%, *P =* 0.039). The referred cases had a seizure before reaching the reference maternity hospital.

**TABLE 3:** Maternal and perinatal outcomes of women with preeclampsia, according to the type of hospitalization.

Maternal and perinatal outcomes	External referrals	Direct admissions	*p*‐value
	(*N* = 37)	(*N =* 391)	
Spontaneous labor	4 (10.8%)	43 (11.0%)	1.00
Route of delivery			
Cesarean section	30 (81.1%)	283 (72.4%)	0.34
Vaginal delivery	7 (18.9%)	108 (27.6%)	
Mean time between admission and delivery (days) (SD)	1.68 (2.35)	1.94 (2.65)	0.49
Mean length of hospital stay (days) (SD)	5.49 (3.05)	5.58 (3.27)	0.99
Moment of PE diagnosis			
Pregnancy or childbirth	36 (97.3%)	362 (92.5%)	0.52
Postpartum	1 (2.7%)	29 (7.4%)	
Moment of diagnosis of severe features^a^			0.21
Pregnancy or childbirth	32 (97.0%)	156 (88.6%)	
Postpartum	1 (3.0%)	20 (11.4%)	
Mean GA (weeks) at diagnosis of PE with severe features (SD)^b^	33.3 (3.45)	34.2 (3.79)	0.09
Mean GA at childbirth (weeks)^c^	33.6 (3.27)	36.1 (2.97)	<0.001
Prematurity^c^	33 (89.2%)	151 (38.8%)	<0.001
Use of magnesium sulfate^a^	33 (89.2%)	175 (44.8%)	<0.001
Eclampsia	2 (5.4%)	2 (0.5%)	0.039
Corticosteroids for fetal lung maturation^d^	7 (58.3%)	48 (80.0%)	0.15
Stillbirth^e^	2 (5.3%)	5 (1.2%)	0.11
Neonatal death^e^	3 (8.3%)	8 (1.9%)	0.05
5‐min Apgar <7^e^	5 (13.9%)	17 (4.1%)	0.03
Mean birthweight (SD)^e^	2150 (946)	2730 (800)	<0.001
Birthweight adequacy to gestational age^e^			
SGA	14 (38.9%)	71 (17.1%)	0.008
AGA	20 (55.6%)	295 (71.1%)	
LGA	2 (5.6%)	44 (11.8%)	
Admission to the neonatal ICU^e^	26 (72.2%)	144 (34.9%)	<0.001
Maternal discharge with antihypertensive treatment	34 (91.9%)	255 (65.2%)	0.002

*Note*: Missing: ^c^2. ^b^N considered: 188 (cases of exclusive postpartum severity and cases of preeclampsia without signs of severity were excluded). ^ab^N considered: 209 (cases where there was no severity were excluded). ^d^N considered: 69 (those who had childbirth before 34 weeks). ^e^Number of fetuses: 458 and newborns: 451.

Abbreviations: AGA, adequate for gestational age; ICU, intensive care unit; GA, gestational age; LGA, large for gestational age; PE, preeclampsia; SD, standard deviation; SGA, small for gestational age.

Upon admission, both groups had low frequency of spontaneous labor (10.8% among external referrals and 11.0% in direct admissions, *P* = 1.00). The final route of birth was cesarean section in 81.1% of the external referrals and 72.4% of the direct admissions group (*P* = 0.34). The mean time between admission and childbirth (1.68 ± 2.35 days among external referrals and 1.94 ± 2.65 days among direct admissions, *P* = 0.49) and the mean total length of hospital stay (roughly 5 days) were similar in both groups. However, the GA at childbirth differed significantly: 33.6 ± 3.27 weeks among external referrals and 36.1 ± 2.97 weeks among direct admissions (*P* < 0.001). Among newborns born to referred women, a 5‐min Apgar score below 7 was over three times more frequent (13.9 vs. 4.1%, *P* = 0.03). The birthweight was lower (2150 ± 946 g vs. 2730 ± 800 g, *P* < 0.001), with greater proportion of small for GA infants (38.9 vs. 17.1%, *P* = 0.008), and the frequency of admission to the neonatal ICU was higher (72.2% vs. 34.9%, *P* < 0.001). There were no maternal deaths, and there was no difference in perinatal deaths between groups (Table 3).

Post‐hoc analyses were performed to ascertain that the differences observed in maternal and perinatal outcomes were associated (or not) with the severity of PE or prematurity. The subgroup analysis of preeclampsia with severe features according to modality of admission exhibited a similar pattern to the one presented in Table 3, except for not showing difference in 5‐min Apgar scores and in birthweight adequacy to GA. The prematurity analysis showed that 5‐min Apgar scores, birthweight, birthweight adequacy to GA, and admission to neonatal ICU were not significant. These analyses can be found in Tables S1 and S2.

The multivariate logistic regression was performed comparing any adverse maternal outcome and social, demographic, and clinical possible predictors. The logistic regression is shown in Table 4. Prematurity could not be included in the model since it was 100% present among the external referrals with adverse maternal outcomes. The variables independently associated with adverse maternal outcome were external referral, with odds ratio (OR) 13.24 and 95% confidence interval (CI) 1.79–1690.84, maternal age >35 years (OR 2.18, 95% CI 1.03–5.14), chronic hypertension (OR 2.51, 95% CI 1.17–5.85), and use of antihypertensive drugs during pregnancy (OR 1.95, 95% CI 1.09–3.58).

**TABLE 4:** Multivariable binary logistic regression of the occurrence of any adverse maternal outcome.

Variable	OR	95% CI	*p*‐value
External referral	13.24	1.79–1690.84	0.005
Maternal age >35 years	2.18	1.03–5.14	0.04
Skin color			
Pardo	0.75	0.41–1.39	0.35
Black	0.71	0.27–2.191	0.53
Skin color black			
Schooling			
Higher education or postgraduation	0.86	0.42–1.85	0.69
Primigravida	1.03	0.58–1.83	0.92
Diabetes	1.12	0.63–2.02	0.70
Chronic hypertension	2.51	1.17–5.85	0.02
Multiple pregnancy	1.25	0.46–4.15	0.68
Use of antihypertensive drugs during pregnancy	1.95	1.09–3.58	0.02

Abbreviations: CI, confidence interval; OR, odds ratio.

Finally, a descriptive analysis of delays in health care that happened at the lower complexity settings is shown in Table 5.

**TABLE 5:** Descriptive analysis of delays in health care of women with preeclampsia at the lower complexity settings.

Healthcare delay marker	Overall (*N* = 37)
Mean distance (km) from the lower complexity center to the tertiary center (SD)^a^	58.9 (48.1)
Mean time (h) from requesting referral to admission at the tertiary center (SD)^a^ ^,b^	5.14 (4.16)
Use of magnesium sulfate	
Happened at the lower complexity setting	25 (67.6%)
It did not happen at the lower complexity setting, but happened at the tertiary center	8 (21.6%)
No use of magnesium sulfate whatsoever	4 (10.8%)
Assessment of fetal well‐being at the lower complexity center	
Fetal heart rate	29 (78.4%)
Cardiotocography	14 (37.8%)
Obstetric Doppler	3 (8.1%)

^a^ Cases that were excluded from this analysis because they were referred from within the same hospital complex (another building).

Missing data: ^b^6.

## 4 DISCUSSION

Overall, the referred cases had greater severity than the standard admissions. This study showed an association between the admission modality and the GA at the diagnosis of PE with severe features, as well as the GA at childbirth. In both cases, the GA was lower among pregnant women referred compared to those admitted for childbirth directly at the tertiary hospital. Referrals also correlated with lower birthweight, small for GA newborns, higher frequency of 5‐min Apgar score below 7, and higher rates of admission to neonatal ICU.

Preeclampsia burdens 2 to 4% of all pregnancies globally.^3^ The present study found overall prevalence of 10.9%, exceeding previous data from the same maternity hospital, which reported a prevalence of 7.3%.^15^ Moreover, PE is a leading cause of severe maternal morbidity and mortality globally. A Brazilian multicenter study found that 70% of the hospitalizations for severe maternal morbidity were due to hypertension.^16^ Among these, there were 8.3 cases of maternal near miss for every maternal death. A maternal near miss is defined when a woman nearly dies from a severe condition during pregnancy, childbirth, or 42 days postpartum but ultimately survives.^17^ Concerning maternal deaths, a recent systematic analysis from maternal deaths from 2009 to 2020 found that hypertensive disorders were the third most common cause of maternal mortality in the world and the main cause in Latin America.^18^

The administration of magnesium sulfate for women with severe PE is the main medical intervention for avoiding adverse outcomes.^4^ However, a previous study evaluated the availability of magnesium sulfate in the municipality where the maternity hospital is located and found that, despite legislation and protocols affirming the need for magnesium sulfate, this drug was only administered in maternity hospitals. Another interesting finding was that magnesium sulfate was unavailable in primary care facilities.^9^ Thus, it is arguable that the healthcare system builds access barriers that constitute delays in phases II and III, when it is difficult to access healthcare facilities and receive adequate care, respectively.^7^

Most of this burden could be prevented with opportune healthcare provision, observing signs of severity, and evaluating transfer to a higher care facility when necessary is considered.^19^ In countries like Brazil, where the public health system is the main source of health care, every facility is responsible for a different complexity level.^20^ Because secondary and tertiary level facilities in the public health system are limited, there must be an organized network of referrals within the system.^21^

In the Brazilian state of São Paulo, in the Southeast region, where the present research was conducted, a decree from 2010 established the Healthcare Services Offer Regulation Center (from the acronym in Portuguese: Central de Regulação de Ofertas de Serviços de Saúde – CROSS).^12^ CROSS controls the offer of the state's public medical assistance according to prespecified geographical regions. The Women's Hospital of UNICAMP is one of the tertiary hospitals CROSS might direct women to after they are evaluated in smaller or lower risk facilities.

This referral system manages outpatient, urgent, and emergent referral requests. The present maternity hospital receives patients from CROSS for the outpatient clinics and urgent and emergent care (such as the ones from this study). One of the most common conditions women from the outpatient clinics have is chronic hypertension. Previous data from the same maternity hospital showed that 40% of the women from the outpatient clinic who had chronic hypertension developed superimposed PE. In this cohort, superimposed PE was associated with worse outcomes, such as cesarean sections, low birthweight, and admission to the neonatal ICU.^22^, ^23^ Most importantly, the frequency of early‐onset PE (diagnosis before 34 weeks) was 40%. It is known that early‐onset PE is more severe than late‐onset PE.^4^

Early recognition and management of PE could avoid maternal and neonatal morbidity because PE is a progressive disease.^19^ In this study, 89.2% of the referred women had PE with severe features. Contrastingly, 44.8% of the women that were admitted directly at the university hospital developed signs of severity. Late recognition of PE might explain the greater proportion of severe cases leading to earlier GA at childbirth and lower birthweight, outcomes associated with PE and major drivers of neonatal ICU admissions.^3^, ^24^, ^25^ Further, fetal growth restriction (FGR) is strongly connected to PE because of the placental dysfunction. FGR and prematurity could explain the threefold increase in low 5‐min Apgar scores among referred women. Although there might be overlaps between severity of preeclampsia and preterm birth in terms of association with poor outcomes, because preeclampsia is a progressive disease, it is most likely that the ideal timing of the diagnosis of preeclampsia was frequently missed among referred cases; otherwise, the outcomes would have been similar to the outcomes of directly admitted patients.

Finally, because perinatal management relies on GA and fetal anatomy, a review of the fetal and neonatal deaths from this cohort showed that all of the fetal deaths occurred in pregnancies with PE and severe features, ranging from 22 to 35 weeks of GA. Among these, most of them were direct admissions, including the most severe cases, diagnosed at 22 and 23 weeks of GA. Regarding the neonatal deaths, eight occurred among newborns whose mothers were directly assisted by the tertiary hospital; however, four (50%) were newborns with complex malformations or confirmed aneuploidies, so the neonatal death might not have been associated with PE. This review reinforces the complexity of cases this maternity hospital manages. Therefore, the availability of hospital beds should be a concern and implies the need for a regulation system with established criteria for referrals.

It is noteworthy that, after being assisted at the reference hospital, all women eventually return to their original primary care providers, where the clinical assessment for hypertension and future risk of cardiovascular diseases should be performed. However, it is noted that this care is often ineffective, despite the risks of PE at short, medium, and long‐term periods. For instance, there is a twofold increase for a broad range of cardiovascular diseases after term PE, and the risk is even higher with severe forms of the disease (PE and preterm delivery, recurrent PE, and FGR).^26^, ^27^ The risks of PE after the pregnancy–postpartum period also encompass a fourfold risk of microalbuminuria, a marker of initial kidney injury, and end‐stage kidney injury, dyslipidemia, and diabetes mellitus.^27^

The inadequate care was observed on a Brazilian cross‐sectional study of the postpartum care of women with PE, where 96% of the women had a scheduled visit at their primary care provider in Brazil.^27^ However, 65.3% of them had their blood pressure measured, 34.7% received counseling about lifestyle habits, and 1.3% of them were made aware of the long‐term risks of PE, thus reinforcing the need for better qualifications of primary care providers.

The strengths of the present study lie in the detailed report of the referral system for obstetric emergencies and the demonstration that even severe cases of PE can be managed and result in good maternal and perinatal outcomes if the tertiary center is of good quality. However, this study also has limitations, including its retrospective and single‐center design and the small number of referred cases given the restrictions imposed by the pandemic. Nevertheless, it could be used as an initial approach to a complex issue that should be addressed in future studies.

## 5 CONCLUSION

Preeclampsia was associated with worse maternal and perinatal outcomes among referred women. The healthcare access regulation system should be organized and include practical tools such as fullPIERS to aid in managing referrals. Nevertheless, quality antenatal care plays a key role in adequate and opportune diagnosis of pregnancy complications.

## AUTHOR CONTRIBUTIONS

Juliana da‐Costa‐Santos: I declare that I participated in the conception of the study design, data collection, statistical analysis, manuscript writing and that I have seen and approved of the final version. I have the following conflicts of interest: None. Guilherme F. Borges: I declare that I participated in conception of the study design, data collection, manuscript writing and that I have seen and approved of the final version. I have the following conflicts of interest: None. Vitor L. V. Reis: I declare that I participated in the data collection, manuscript writing and that I have seen and approved of the final version. I have the following conflicts of interest: None. Rodolfo R. Japecanga: I declare that I participated in the data collection, manuscript writing and that I have seen and approved of the final version. I have the following conflicts of interest: None. Maykon A. Carvalho: I declare that I participated in the data collection, manuscript writing and that I have seen and approved of the final version. I have the following conflicts of interest: None. Ana Julia G. Oliveira: I declare that I participated in the data collection, manuscript writing and that I have seen and approved of the final version. I have the following conflicts of interest: None. Laura C. Vinchi: I declare that I participated in the data collection, manuscript writing and that I have seen and approved of the final version. I have the following conflicts of interest: None. Arthur B. P. Righi: I declare that I participated in the ethical approval obtaining, data collection, manuscript writing and that I have seen and approved of the final version. I have the following conflicts of interest: None. Jussara Mayrink: I declare that I participated in the conception of the study design, manuscript writing and that I have seen and approved of the final version. I have the following conflicts of interest: None. José Paulo Guida: I declare that I participated in the ethical approval obtaining, conception of the study design, manuscript writing and that I have seen and approved of the final version. I have the following conflicts of interest: None. Renato T. Souza: I declare that I participated in the manuscript writing and that I have seen and approved of the final version. I have the following conflicts of interest: None. Fernanda G. Surita: I declare that I participated in the manuscript writing and that I have seen and approved of the final version. I have the following conflicts of interest: None. José G. Cecatti JG: I declare that I participated in the manuscript writing and that I have seen and approved of the final version. I have the following conflicts of interest: None. Maria Laura Costa: I declare that I participated in the ethical approval obtaining, conception of the study design, statistical analysis, manuscript writing and that I have seen and approved of the final version. I have the following conflicts of interest: None.

## FUNDING INFORMATION

JCS was financed in part by the Coordenação de Aperfeiçoamento de Pessoal de Nível Superior—Brasil (CAPES)—Finance Code 001 (process 88887.896655/2023–00). MAC was funded in part by the Programa Institucional de Bolsas de Iniciação Científica—PIBIC/CNPq–UNICAMP. AJGO was financed, in part, by the São Paulo Research Foundation (FAPESP), Brasil. Process Number #2023/13456‐4. MLC was supported by Fundação de Amparo à Pesquisa do Estado de São Paulo (FAPESP) [grant number 2021/09937–1] and by Conselho Nacional de Desenvolvimento Científico e Tecnológico (CNPq) [grant number 408407/2021–2].

## CONFLICT OF INTEREST STATEMENT

The authors have no conflicts of interest to declare.

## Supporting information


**Table S1.** Maternal and perinatal outcomes of women with preeclampsia with severe features, according to the type of hospitalization.

**Table S2.** Perinatal outcomes of preterm newborns after a live birth from women with preeclampsia, according to the type of hospitalization.

## Data Availability

The participants of this study did not give written consent for their data to be shared publicly, so due to the sensitive nature of the research, supporting data is not available.
