# Supplementary material for: Direct versus referred admission to the maternity hospital due to preeclampsia: Does it influence pregnancy outcomes?
Source: Int J Gynaecol Obstet. 2026 Jan 5;173(3):1528–36. doi: 10.1002/ijgo.70772 (PMC13173636; doi:10.1002/ijgo.70772)
Supplement: Supplementary file 1 — Table S1. Maternal and perinatal outcomes of women with preeclampsia with severe features, according to the type of hospitalization. [file IJGO-173-1528-s001.docx]

**Supplementary table 1 – Maternal and perinatal outcomes of women with preeclampsia with severe features, according to the type of hospitalization.**

| **Preeclampsia with severe features** | **External referrals (N=32)** | **Direct admissions (N=156)** | **p value** |  | **P** |  |
| --- | --- | --- | --- | --- | --- | --- |
| **Mean GA at childbirth (weeks) ^a^** | 33.5 (3.41) | 35.1 (3.48) | 0.001 |  |  |  |
| **Route of delivery** |  |  | 1.000 |  |  |  |
| Cesarean section | 25 (78.1%) | 123 (78.8%) |  |  |  |  |
| Vaginal delivery | 7 (21.9%) | 33 (21.2%) |  |  |  |  |
| **Mean time between admission and delivery (days) (SD)** | 1.53 (1.63) | 2.17 (2.83) | 0.354 |  |  |  |
| **Mean length of hospital stay (days) (SD)** | 5.41 (2.73) | 6.33 (3.28) | 0.170 |  |  |  |
| **Prematurity ^a^** | 29 (90.6%) | 83 (53.9%) | 0.0002 |  |  |  |
| **Neonatal death ^b^** | 3 (9.7%) | 5 (3.2%) | 0.128 |  |  |  |
| **5^th^ minute Apgar < 7 ^b^** | 4 (12.9%) | 12 (7.7%) | 0.309 |  |  |  |
| **Mean birthweight (SD) ^b^** | 2120 (916) | 2530 (873) | 0.012 |  |  |  |
| **Birthweight adequacy to gestational age ^b^** |  |  | 0.101 |  |  |  |
| SGA | 12 (38.7%) | 33 (21.2%) |  |  |  |  |
| AGA | 18 (58.1%) | 109 (69.9%) |  |  |  |  |
| LGA | 1 (3.2%) | 14 (9.0%) |  |  |  |  |
| **Admission to the neonatal ICU ^b^** | 23 (74.2%) | 80 (51.3%) | 0.031 |  |  |  |

Missing data: ^a^: 2.

^b^ N considered: 187 newborns (2 of the 32 referred women had fetal deaths, and 1/32 had twins). This contrasts with the 188 women considered for the first part of this analysis.

SD: standard deviation. SGA: small for gestational age. AGA: adequate for gestational age. LGA: large for gestational age. ICU: intensive care unit.
