# Supplementary material for: Direct versus referred admission to the maternity hospital due to preeclampsia: Does it influence pregnancy outcomes?
Source: Int J Gynaecol Obstet. 2026 Jan 5;173(3):1528–36. doi: 10.1002/ijgo.70772 (PMC13173636; doi:10.1002/ijgo.70772)
Supplement: Supplementary file 2 — Table S2. Perinatal outcomes of preterm newborns after a live birth from women with preeclampsia, according to the type of hospitalization. [file IJGO-173-1528-s002.docx]

**Supplementary table 2 – Perinatal outcomes of preterm newborns after a live birth from women with preeclampsia, according to the type of hospitalization.**

| **Live preterm newborns** | **External referrals (N=32)** | **Direct admissions (N=171)** | **p value** |
| --- | --- | --- | --- |
| **Neonatal death** | 3 (9.4%) | 6 (3.5%) | 0.153 |
| **5^th^ minute Apgar < 7** | 5 (15.6%) | 14 (8.2%) | 0.190 |
| **Mean birthweight (SD)** | 1980 (825) | 2120 (761) | 0.353 |
| **Birthweight adequacy to gestational age** |  |  | 0.217 |
| SGA | 14 (43.8%) | 48 (28.1%) |  |
| AGA | 17 (53.1%) | 111 (64.9%) |  |
| LGA | 1 (3.1%) | 12 (7.0%) |  |
| **Admission to the neonatal ICU** | 27 (84.4%) | 121 (70.8%) | 0.132 |

SD: standard deviation. SGA: small for gestational age. AGA: adequate for gestational age. LGA: large for gestational age. ICU: intensive care unit.
